# Studying the Relation Between Fibromyalgia Severity and Neutrophil-to-Lymphocyte Ratio, Platelet-to-Lymphocyte Ratio, and Mean Platelet Volume

**DOI:** 10.7759/cureus.24847

**Published:** 2022-05-09

**Authors:** Acchudha Krishnan Rajaram Jayakrishnan, Sekhar V Easwar, Joslyn Thattil, Mani Vignesh, Sudeep Rath, Abhinav Prithvi, Vishal Marwaha, Mithun CB, Sandeep Surendran

**Affiliations:** 1 Rheumatology, Amrita Institute of Medical Sciences, Amrita Vishwa Vidyapeetham, Kochi, IND

**Keywords:** neutrophil to lymphocyte ratio (nlr), visual analogue scale, mean platelet volume, neuroinflammation, fibromyalgia

## Abstract

Background: Fibromyalgia is characterized by chronic widespread pain, which has been linked to neuroinflammation. Hematological indices, i.e., neutrophil-to-lymphocyte ratio (NLR), platelet-to-lymphocyte ratio (PLR), and mean platelet volume (MPV) have been shown to be effective markers in neurological diseases like depression.

Aims: To study the association between fibromyalgia severity and the hematological indices (NLR, PLR, and MPV).

Subjects and Methods: This was a hospital-based cross-sectional study of fibromyalgia patients satisfying the 2016 modification of the 2010/11 ACR criteria. Demographic and clinical characteristics were recorded along with fibromyalgia outcomes and hematological indices. Statistical analysis was done using descriptive statistics, ROC analysis using the Youden index, and Pearson and Spearman correlations.

Results: A total of 266 patients were recruited. The (mean ± S.D) NLR, MPV, and PLR were 1.92 ± 1.26, 8.94 ± 1.25, and 119.48 ± 76.91, respectively. Patients with severe visual analog scale (VAS) pain scores had lower MPV (8.8 ± 1.3) than those with mild/moderate pain (9.2 ± 1.1, p = 0.016). MPV showed a mild negative correlation with the Fibromyalgia Impact Questionnaire-Revised (FIQR) score (R2 -0.31 p 0.004). MPV threshold of 8.95 was discriminated severely from mild/moderate VAS-pain score with a sensitivity of 52.3 % and specificity of 66.7%.

Conclusions: MPV can possibly be considered as a biomarker for predicting pain severity in fibromyalgia. Given its inexpensive nature, MPV can be used as a cost-effective method to assess fibromyalgia severity in rural India.

## Introduction

Fibromyalgia typically presents as chronic widespread musculoskeletal pain syndrome and is often accompanied by fatigue, insomnia, cognitive disturbances, and multiple somatic symptoms. It occurs more commonly in women with its prevalence in different countries varying between 1-3% of the general population. The exact mechanisms that lead to the development of fibromyalgia are unknown but the key etiopathology mechanisms are alterations in cortical and spinal pain processing along with small fiber neuropathy [1]. There is evidence to suggest that these mechanisms are malactivated due to neuro-inflammatory changes [2]. Systematic review and metanalyses have shown that pro-inflammatory cytokines, interleukin-6 (IL6) and interleukin-8 (IL8), are found to be elevated in fibromyalgia [3]. Studies have also shown these pro-inflammatory cytokines, especially IL-8, tend to correlate with pain and other outcome measures of disease severity [4]. However, to do serum cytokine analysis in all fibromyalgia patients is not practically feasible in India as such facilities are only available in certain tertiary care centers and apex academic institutes [5].

Increasingly, the role of non-traditional hematological indices in identifying systemic inflammation is being studied. The neutrophil-to-lymphocyte ratio (NLR), platelet-to-lymphocyte ratio (PLR), and mean platelet volume (MPV) are three of the most commonly studied hematological indices with higher NLR, PLR, and MPV values being suggestive of underlying inflammation. These indices are cost-effective and readily available in all patients [6].

There are few studies that have studied these hematological studies in fibromyalgia and, to the best of our knowledge, no previous studies have studied the relation between NLR, PLR, and MPV with fibromyalgia disease severity. The aim of our study was to investigate associations between fibromyalgia outcome measures and the hematological indices (NLR, PLR, and MPV).

## Materials and methods

Study population and design

The study was a hospital-based cross-sectional study conducted at Amrita Institute of Medical Sciences, Kochi, Kerala, India. The study was approved by the Institutional Ethics Committee of Amrita Institute of Medical Sciences, Kochi, Kerala, India (IRB-AIMS-2020-458), and data were retrospectively collected using hospital electronic medical records for the period of June 2018 to June 2020. The diagnosis of fibromyalgia was made using the 2016 modification of the 2010/11 American College of Rheumatology (ACR) criteria. These were patients seen in the Rheumatology clinic for fibromyalgia. Other possible causes of chronic pain were excluded through clinical and laboratory tests, while the causes of inflammation and infection were ruled out. Patients with type 2 diabetes mellitus hypothyroidism, hyperthyroidism, inflammatory rheumatic disease, and autoimmune disease were not included in the study. Patients with a history of major depression, cerebrovascular events, and malignancy were excluded from the study. Patients were also excluded if they had cardiovascular disease, hypertension, peripheral artery disease, active chronic obstructive lung disease, heart failure, chronic kidney disease, diabetes mellitus, pulmonary or neurological disease, peripheral neuropathy, hepatic disease, or history of alcohol abuse. 

Demographic, biochemical parameters, and disease outcome measures 

Routine demographic and clinical details were assessed for every patient. Hematological parameters were collected from the records. All tests were analyzed using a hematology analyzer, CELL-DYN® 3700 (Abbott Laboratories, Chicago, Illinois, United States), within 30-45 minutes after the blood was collected. Hemoglobin values were expressed in g/dL. Leucocyte, neutrophil, lymphocyte, and platelet counts were recorded and expressed in 10^3/μL. NLR and PLR were calculated using the results of these parameters. To assess fibromyalgia severity, the outcomes used were the visual analog scale (VAS) pain score and Fibromyalgia Impact Questionnaire-Revised (FIQR) score. FIQR is the latest validated composite patient self-reported instrument that assesses the impact of fibromyalgia symptoms and functional impairment. VAS pain score of 6 was considered to be severe pain.

Statistical methods

Descriptive statistics for the constant variables were expressed as mean and SD while the categorical variables were expressed as numbers and percentages. Pearson and Spearman correlation tests were done to determine the relationship between the hematological indices (NLR, PLR, and MPV) with categorical land non-categorical pain scores, respectively. In addition, the Mann-Whitney U test was used to assess the relationship between these indices with the categories. Receiver operating characteristic (ROC) curve analysis was used to investigate for significant indices as to whether it could distinguish between severe pain and mild/moderate pain on the VAS pain scale. Youden's index was assessed to identify the optimum cut-off to distinguish severe from mild/moderate pain. The statistical analyses and graphical plotting were performed using SPSS for Windows, Version 16.0 (Released 2006; SPSS Inc., Chicago, United States), and a p-value of less than 0.05 value was considered to be statistically significant with all statistical tests performed were two-tailed.

## Results

We recruited 266 patients for this study. The baseline demographics along with the disease outcome measures and biochemical indices are shown in Table 1 along with data from two other published studies [7,8]. Table 2 shows the statistical associations between the hematological indices and fibromyalgia disease outcome measures.

**Table 1:** Association of Hematological Indices and Fibromyalgia Disease Outcome Measures NLR: neutrophil-to-lymphocyte ratio; PLR: platelet-to-lymphocyte ratio; MPV: mean platelet volume; FIQR: Fibromyalgia Impact Questionnaire-Revised; VAS: visual analoge scale

Hemogram Index	FIQR		VAS Pain	
	Correlation coefficient	p-value	Correlation coefficient	p-value
NLR	0.004	0.75	0.047	0.441
PLR	-0.031	0.21	-0.037	0.551
MPV	- 0.31	0.004	-0.148	0.016

**Table 2:** Comparative Baseline, Hematological, and Disease Parameters NLR: neutrophil-to-lymphocyte ratio; MPV: mean platelet volume; PLR: platelet-to-lymphocyte ratio; ESR: erythrocyte sedimentation rate; BMI: body mass index; FIQR: Fibromyalgia Impact Questionnaire-Revised; VAS: visual analog scale

Sl #	Parameter	Current Study	Semra et al., 2017 [7]	İlgün et al., 2016 [8]
1	Sample Size	266	197	70
2	Age (years)	41.75 ± 10.2	39.74 ± 7.6	43.4 ± 9.8
3	Sex	258 females, 8 males	168 females, 29 males	56 females, 14 males
4	BMI (kg/m^2^)	26.29 ± 4.4	27.17 ± 17	28.2 ± 5.2
5	NLR	1.92 ±1.256	1.93 ± 0.73	1.9 + 0.6
6	MPV	8.94 ± 1.25	10.46 ± 1.13	-
7	PLR	119.48 ± 76.9	NA	128.5 ± 40.2
8	ESR (mm/hr)	22.30 ± 11.96	14.31 ± 8.16	10.7 ± 5.6
9	VAS Pain	6.35 ± 2.3	NA	NA
10	FIQR	54.89 ± 19.42	NA	NA

**Figure 1:**
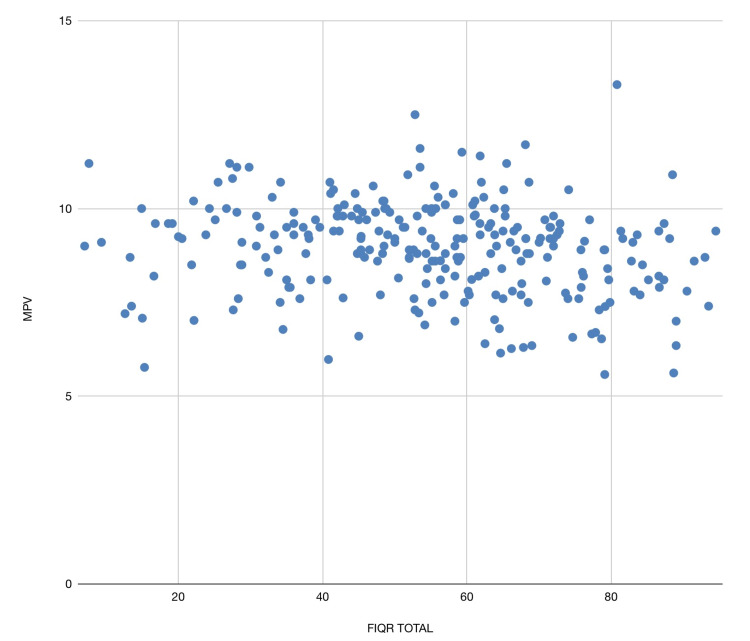
Scatter Plot Showing the Relation Between MPV and FIQR Scores MPV: mean platelet volume; FIQR: Fibromyalgia Impact Questionnaire-Revised

A significant mild negative correlation was found between FIQR score and MPV and is graphically shown in Figure 1. Both NLR and PLR values were similar in patients with severe VAS pain and mild/moderate VAS pain (p value= 0.55 and 0.44, respectively). But patients with severe pain had lower MPV (8.8 ± 1.3) than patients with mild/moderate pain (9.2 ± 1.1) on VAS pain scales (p = 0.016).

ROC analysis was done based on MPV in discriminating severe pain from mild/moderate pain. The AUC of this curve was 0.591 (95%CI 0.519-0.662). From the Youden's index, the optimal MPV threshold of 8.95 was found to discriminate severe pain from mild/moderate pain on the VAS pain score with a sensitivity of 52.3% and specificity of 66.7%.

## Discussion

In this study, we evaluated novel hematological indices (NLR and PLR) along with MPV in fibromyalgia patients and studied their associations with pain severity. Our study shows that of the three indices evaluated, it is MPV that shows associations with the severity of fibromyalgia more than NLR or PLR indices. The MPV showed a mild negative correlation with both FIQR (p=0.004) and reported pain in fibromyalgia. Though the correlation between MPV and VAS pain score is significant (p = 0.016); this relation is not clinically significant. However, it was noted that the absolute MPV value tended to be lower in patients with severe pain of VAS pain scores. This is the first study to the best of our knowledge that has studied the association between hematological indices and pain severity in fibromyalgia.

The association between MPV and pain severity could be related to the cytokine signatures reported in fibromyalgia. We have already mentioned the nature of IL-6 and IL-8 in fibromyalgia syndrome (FMS); however, these cytokines have systemic knock-down effects. IL-8 and IL-6 are associated with platelet hyperactivity and peripheral activation in the nervous system of platelets in fibromyalgia due to the cytokines could lead to a lower MPV centrally. This phenomenon has been reported in other studies on diseases with over systemic inflammation (rheumatoid arthritis, lupus, etc.) where the lower MPV level surrogates an active and/or chronic inflammatory state in the body [9]. In the era of personalized medicine, some of these cytokines may be used as biological markers or in therapeutic stratification [10]. But the expensive and technical nature of such testing limits these strategies to resource-rich settings. Rather than using cytokine signatures in fibromyalgia, our study suggests the MPV could potentially be used as a surrogate marker in assessing disease severity instead of cytokine signatures (especially IL-8). However, as the correlation is only mild, the possibility of multifactorial non-linear inter-relations needs to be considered. Newer analytic methods like machine learning could elucidate these relations and further research is needed for the same.

In comparison to other cohorts, the ranges of NLR, PLR, and MPV reported in our study are comparable (Table 1). Another interesting observation is how the mean MPV in our study is numerically lower than the mean MPV in the other study that reported the same. However, no statistical comparison could be done.

An important domain to consider here is the link between atherosclerotic disease and MPV. It is well known that MPV is raised in atherosclerotic cardiovascular disease and cerebrovascular ischemia. A study by Su et al. points to a significantly increased risk of coronary heart disease in patients with fibromyalgia [11]. Further studies are needed to assess whether MPV can help stratify fibromyalgia patients who are at risk for development of cardiovascular disease [12].

Our study is not, however, without limitations. The retrospective design of the study fails to discount patients who have other causes of hemogram alterations like viral fever, drugs, and smoking. Further prospective studies can assess this flaw. Such studies can simultaneously also assess the cardiovascular risk status and cytokine profiles to improve the clinical relevance.

## Conclusions

From the current study, an inference can be made that there is a relation between MPV and fibromyalgia severity. The other hematological markers do not show any relation with disease severity. The importance of MPV could be linked with IL-8, which is a key factor for both platelet activity and fibromyalgia etiopathology. This study also suggests that MPV is a cost-effective tool that can be utilized to diagnose or stratify the severity of fibromyalgia even in the peripheral centers of rural India. Future long-term follow-up studies can better substantiate this study.
